# Olfactory Stimulation with Japanese Soy Sauce Improves Upper Limb Performance

**DOI:** 10.1155/2019/2748721

**Published:** 2019-10-01

**Authors:** Yutaka Yano, Yoshihiro Murata, Mutsuo Taniguchi, Fumino Okutani, Masahiro Yamaguchi, Hideto Kaba

**Affiliations:** ^1^HITO Hospital, Shikokuchuo, Ehime 799-0121, Japan; ^2^Department of Occupational Therapy, Tosa Rehabilitation College, Kochi 781-5103, Japan; ^3^Department of Physiology, Kochi Medical School, Kochi University, Nankoku, Kochi 783-8505, Japan

## Abstract

**Background:**

We have observed changes in body reactions during cooking, which is one of the treatment modalities used in occupational therapy. The perception of food-related odors during cooking may have behavioral effects on human activities through the activation of appetitive motivation.

**Objectives:**

We investigated whether odor components contained in seasonings could facilitate the human motor system and the specificity of this effect.

**Methods:**

The subjects were 72 healthy adults, randomly assigned to a water exposure group, a phenylethyl alcohol (PEA, pleasant rose-like odor) exposure group, and a Japanese soy sauce (Koikuchi Shoyu) exposure group (*n* = 24 each). The subjects' olfactory sense was stimulated by their sniffing of three different test tubes containing 5 ml of water, PEA, or Japanese soy sauce for 20 sec while they were seated. The modified Functional Reach Test (mFRT), which mimics a functional activity that is required in daily living and assesses a reliable measure of sitting balance, was performed prior to and immediately after the sniffing.

**Results:**

Sniffing the soy sauce increased the subjects' mFRT scores. This facilitation effect was odorant-specific and was absent when the subjects were presented with water or PEA.

**Conclusions:**

Cooking interventions are aimed at improving tool-handling skills such as using knives and chopsticks. The results indicate that treatment interventions using odors of seasonings would be effective for improving subjects' physical functions.

## 1. Introduction

After experiencing a stroke, which is a common cause of long-term disability, many patients with central nervous system (CNS) disorders have difficulty with instrumental activities of daily living (IADLs). Occupational therapists thus work to improve their patients' performance of IADLs so that they can adapt to daily life and social life, with the goal of enhancing their patients' quality of life.

Cooking as an occupational therapy activity is an IADL that is aimed at improving mostly tool-handling skills such as using a knife and chopsticks. Cooking has also been found to be important for individuals' sense of life satisfaction [1] and to be strongly related to a variety of cognitive skills [2]. However, many factors are involved in a patient's cooking performance, including physical-environmental factors (e.g., the design of a kitchen and tools) and other types of environmental factors such as food-related odor cues.

We have noted changes in the physical reactions of patients with CNS system disorders that occurred during cooking interventions. One of the factors contributing to these changes may be food-related odor cues, which are often strong motivators for eating. Chemosensory perception is the result of an interaction between the olfactory and the trigeminal systems. The olfactory system is responsible for the perception of odor qualities, whereas the trigeminal system conveys sensations such as burning, stinging, pungency, temperature, or pain [3, 4]. Neuroimaging studies have shown that stimulation with food-related odors is a potent elicitor of cerebral activity in brain reward circuits, including frontal, ventral striatal, amygdala, and midbrain regions [5, 6]. Compared to nonfood odors, food-related odors generated higher activation in the anterior cingulate cortex, insula, and putamen [7]. We hypothesized that the perception of food-related odors during cooking could modulate the motor system by driving the motivation to eat. Although a number of studies have found beneficial effects of food-related odors on swallowing [8, 9], postural stability [10], gait performance [11], and grasping movement toward a food object [12], most of the food-related odors so far examined in relation to the motor system were trigeminal stimulants such as black pepper and lavender [8–11]. Therefore, the relationship between food-related odors and the motor system has not yet been fully clarified.

We thus first selected Japanese soy sauce (Koikuchi Shoyu) as a powerful appetitive cue used for olfactory stimulation, because this is a signature seasoning in Japanese food culture and is easily obtained in daily life. We also suspected that Japanese subjects are less likely to dislike the smell of soy sauce. We then compared changes in the modified Functional Reach Test (mFRT) score, a reliable measure of sitting balance, in healthy individuals divided into stimulation groups (Japanese soy sauce and phenylethyl alcohol (PEA) with a pleasant rose-like odor) and a nonstimulated group (water).

## 2. Materials and Methods

### 2.1. Subjects

Seventy-two healthy adults who reported normal olfaction and no history of olfactory dysfunction were recruited from the Department of Rehabilitation, HITO Hospital, and we randomly assigned them to a water exposure group, a PEA exposure group, and a Japanese soy sauce exposure group (24 individuals in each group).  Table 1 shows the baseline characteristics of the three groups. There were no significant differences in the sex ratio, mean age, or mean height among the groups.

All subjects gave their informed consent to participate in the study, which was approved by the Ethical Committee of HITO Hospital and performed in accordance with the Declaration of Helsinki (1964).

### 2.2. Olfactory Stimulation

Olfactory stimulation was carried out for each subject in a seated position 30-60 min before lunch or dinner. The subject sniffed a test tube containing 5 ml solution of water, PEA, or Japanese soy sauce for 20 sec. The soy sauce used for the olfactory stimulation was authentically brewed dark-colored soy sauce (Koikuchi Shoyu, Kikkoman, Tokyo). The water was mineral water. The PEA (Wako Pure Chemical Industries, Osaka, Japan) was diluted 100-fold with propylene glycol (Wako).

### 2.3. Assessing Movement and Posture

The mFRT, which mimics a very functional activity that is required in daily living and assesses sitting balance and upper limb motor function, was administered as described by Lynch et al. [13]. Once the subject was seated comfortably on a chair, a yardstick was placed along the subject's shoulder at the level of the acromion. The subject's hips, knees, and ankles were positioned with 90° of flexion. The maximum distance forward that the subject could reach was measured with the upper extremity flexed to 90°. To record the mFRT score, we used an FRT measure (GB–200, OG Wellness, Morioka, Japan). All 72 subjects underwent the FRT measurement both before and immediately after the sniffing protocol. We recorded all measuring processes using a digital video camera (DCR-TRV30, Sony, Tokyo) in order to clarify the accuracy of measurement.

### 2.4. Statistical Analysis

Data were tested for normality (Shapiro-Wilk test) and equal variance (Bartlett test) first. Because the distributions of mFRT scores did not follow a normal distribution, they were analyzed by the nonparametric Wilcoxon signed-rank test for each group. Significance was accepted at *P* < 0.05 (two-tailed). Statistical analyses were performed using JMP software (ver. 11.0; SAS Institute, Cary, NC).

## 3. Results and Discussion

**Figure 1:**
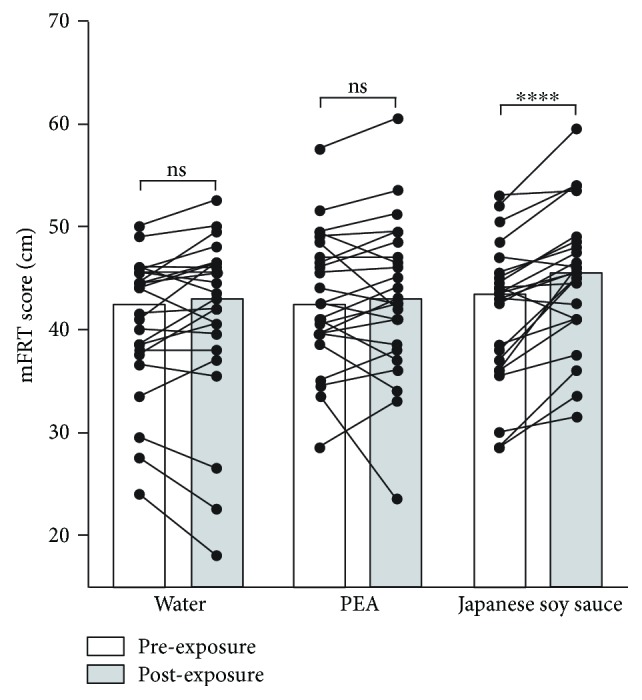
Effects of sniffing water, PEA, or Japanese soy sauce on the mFRT score in healthy adults. Bar graphs indicate the median value of each group. ns: not significant. ^∗∗∗∗^*P* < 0.0001.

Sniffing water or PEA had no effect on the mFRT score (*P* = 0.24 for water, *P* = 0.42 for PEA, *n* = 24, Wilcoxon signed-rank test; Figure 1). In contrast, sniffing Japanese soy sauce significantly increased the subjects' mFRT score (*P* < 0.0001, *n* = 24, Wilcoxon signed-rank test; Figure 1), indicating that the facilitation effect of Japanese soy sauce on the mFRT score was odorant-specific.

To our knowledge, the present data are the first indicating that sniffing Japanese soy sauce improves upper limb performance in healthy individuals. This effect was specifically dependent upon the characteristics of the sniffed soy sauce, being absent with PEA, a pleasant rose-like odor.

Regarding the selectivity of olfactomotor facilitation, Ebihara et al. [8] showed that black pepper odor improved the swallowing reflex in dysphagic older adults, whereas lavender oil did not, suggesting that olfactomotor facilitation's effect on swallowing is odorant-specific. This is in contrast to the observation [9] that both black pepper and lavender improved postural stability in older adults. In agreement with the observation [14] that motivation to eat modulated the neural activity in the mirror-neuron system, facilitating the preparation or the intention to act, Rossi et al. [12] indicated that sniffing food-related odors increased the motor potentials evoked in hand muscles by transcranial magnetic stimulation of the motor cortex. Notably, the odor-induced motor facilitation took place only in cases of congruence between the sniffed odor and the observed grasped food and specifically involved the muscle acting as the prime mover for the hand/fingers shaping in the observed action, revealing complex olfactory cross-modal effects on the human motor system.

Many investigations on the volatile constituents of Japanese soy sauce have been performed in the past, and today, nearly 300 compounds have been identified. Among them, 5(or 2)-ethyl-4-hydroxy-2(or 5)-methyl-3(2*H*)-furanone (4-HEMF) and 4-hydroxy-2,5-dimethyl-3(2*H*)-furanone (4-HDMF) were proposed to be the key odorants in Koikuchi Shoyu [15]. It would be interesting to test whether stimulation with Koikuchi Shoyu or its key odorants is a potent elicitor of cerebral activity in brain reward circuits.

What is the route through which motivation for Japanese soy sauce modulates the activities of trunk muscles involved in sitting balance? The nucleus accumbens, a key structure mediating motivational and emotional processes, has functional connectivity with the primary motor cortex [16], the major contributor to the corticospinal tract, and it projects to the ventral pallidum and the substantia nigra pars reticulata, which control the activity of the midbrain locomotor region/pedunculopontine tegmental nucleus (PPN) [17]. The PPN acts as an interface device between the corticobasal ganglia circuit and the corticocerebellar circuit [18]. The PPN projects to the pontine and medullary reticular formation, which in turn controls reticulospinal pathways involved in locomotion and postural control [17, 19]. The nucleus accumbens also interacts with hypothalamic and limbic structures, such as the amygdala and lateral hypothalamus, a motivation-cognition interface in the control of feeding behavior, either directly or indirectly. Further studies are needed to elucidate the details of how food-related odors as strong motivators of eating interact with the motor system.

## 4. Conclusions

Cooking interventions are used in therapeutic and rehabilitative settings. The present study is the first to demonstrate that olfactory stimulation with Japanese soy sauce, a signature seasoning in Japanese food culture, improves upper limb performance in healthy individuals, emphasizing the importance of cooking interventions for improving instrumental activities of daily living.

## Figures and Tables

**Table 1:** Baseline characteristics of the three groups.

	Water	PEA	Japanese soy sauce	*χ* ^2^/ANOVA
No.	24	24	24	—
Male/female	10/14	11/13	12/12	*χ* ^2^_(2)_ = 0.34, *P* = 0.85
Age (yrs)	26.0 ± 0.7	25.4 ± 0.6	26.9 ± 0.8	*F*_(2, 69)_ = 0.96, *P* = 0.39
Height (cm)	161.5 ± 1.7	161.0 ± 1.8	162.4 ± 1.7	*F*_(2, 69)_ = 0.15, *P* = 0.86

Values are the mean ± SEM.

## Data Availability

Data are available from Yutaka Yano (otrc.yano@tosareha.ac.jp).

## References

[1] Johnston M. V., Goverover Y., Dijkers M. (2005). Community activities and individuals’ satisfaction with them: quality of life in the first year after traumatic brain injury. *Archives of Physical Medicine and Rehabilitation*.

[2] Yantz C. L., Johnson-Greene D., Higginson C., Emmerson L. (2010). Functional cooking skills and neuropsychological functioning in patients with stroke: an ecological validity study. *Neuropsychological Rehabilitation*.

[3] Hummel T. (2000). Assessment of intranasal trigeminal function. *International Journal of Psychophysiology*.

[4] Kollndorfer K., Kowalczyk K., Frasnelli J. (2015). Same same but different. Different trigeminal chemoreceptors share the same central pathway. *PLoS One*.

[5] Berridge K. C. (1996). Food reward: brain substrates of wanting and liking. *Neuroscience & Biobehavioral Reviews*.

[6] Beaver J. D., Lawrence A. D., van Ditzhuijzen J., Davis M. H., Woods A., Calder A. J. (2006). Individual differences in reward drive predict neural responses to images of food. *Journal of Neuroscience*.

[7] Sorokowska A., Schoen K., Hummel C., Han P., Warr J., Hummel T. (2017). Food-related odors activate dopaminergic brain areas. *Frontiers in Human Neuroscience*.

[8] Ebihara T., Ebihara S., Maruyama M. (2006). A randomized trial of olfactory stimulation using black pepper oil in older people with swallowing dysfunction. *Journal of the American Geriatrics Society*.

[9] Ebihara S., Kohzuki M., Sumi Y., Ebihara T. (2011). Sensory stimulation to improve swallowing reflex and prevent aspiration pneumonia in elderly dysphagic people. *Journal of Pharmacological Sciences*.

[10] Freeman S., Ebihara S., Ebihara T. (2009). Olfactory stimuli and enhanced postural stability in older adults. *Gait & Posture*.

[11] Ebihara S., Nikkuni E., Ebihara T., Sakamoto Y., Freeman S., Kohzuki M. (2012). Effects of olfactory stimulation on gait performance in frail older adults. *Geriatrics & Gerontology International*.

[12] Rossi S., De Capua A., Pasqualetti P. (2008). Distinct olfactory cross-modal effects on the human motor system. *PLoS One*.

[13] Lynch S. M., Leahy P., Barker S. P. (1998). Reliability of measurements obtained with a modified functional reach test in subjects with spinal cord injury. *Physical Therapy*.

[14] Cheng Y., Meltzoff A. N., Decety J. (2007). Motivation modulates the activity of the human mirror-neuron system. *Cerebral Cortex*.

[15] Kaneko S., Kumazawa K., Nishimura O. (2012). Comparison of key aroma compounds in five different types of Japanese soy sauces by aroma extract dilution analysis (AEDA). *Journal of Agricultural and Food Chemistry*.

[16] Nishimura Y., Onoe H., Onoe K., Morichika Y., Tsukada H., Isa T. (2011). Neural substrates for the motivational regulation of motor recovery after spinal-cord injury. *PLoS One*.

[17] Takakusaki K. (2017). Functional neuroanatomy for posture and gait control. *Journal of Movement Disorders*.

[18] Mori F., Okada K. I., Nomura T., Kobayashi Y. (2016). The pedunculopontine tegmental nucleus as a motor and cognitive interface between the cerebellum and basal ganglia. *Frontiers in Neuroanatomy*.

[19] Tattersall T. L., Stratton P. G., Coyne T. J. (2014). Imagined gait modulates neuronal network dynamics in the human pedunculopontine nucleus. *Nature Neuroscience*.

